# Exploiting sparseness in *de novo *genome assembly

**DOI:** 10.1186/1471-2105-13-S6-S1

**Published:** 2012-04-19

**Authors:** Chengxi Ye, Zhanshan Sam Ma, Charles H Cannon, Mihai Pop, Douglas W Yu

**Affiliations:** 1 Ecology & Evolution of Plant-Animal Interaction Group, Xishuangbanna Tropical Botanic Garden, Chinese Academy of Sciences, Menglun, Yunnan 666303 China; 2 Ecology, Conservation, and Environment Center; State Key Laboratory of Genetic Resources and Evolution, Kunming Institute of Zoology, Chinese Academy of Sciences, Kunming, Yunnan 650223 China; 3 Department of Computer Science and Center for Bioinformatics and Computational Biology, Institute for Advanced Computer Studies, University of Maryland, College Park, MD, USA; 4 Computational Biology and Medical Ecology Lab; State Key Laboratory of Genetic Resources and Evolution, Kunming Institute of Zoology, Chinese Academy of Sciences, Kunming, Yunnan 650223 China; 5 Ecological Evolution Group, Xishuangbanna Tropical Botanic Garden, Chinese Academy of Sciences, Menglun, Yunnan 666303 China; 6 Department of Biological Sciences, Texas Tech University, Lubbock, TX 79410 USA; 7 School of Biological Sciences, University of East Anglia, Norwich, Norfolk NR47TJ UK

## Abstract

**Background:**

The very large memory requirements for the construction of assembly graphs for *de novo *genome assembly limit current algorithms to super-computing environments.

**Methods:**

In this paper, we demonstrate that constructing a sparse assembly graph which stores only a small fraction of the observed *k-*mers as nodes and the links between these nodes allows the *de novo *assembly of even moderately-sized genomes (~500 M) on a typical laptop computer.

**Results:**

We implement this sparse graph concept in a proof-of-principle software package, *SparseAssembler*, utilizing a new sparse *k-*mer graph structure evolved from the *de Bruijn *graph. We test our *SparseAssembler *with both simulated and real data, achieving ~90% memory savings and retaining high assembly accuracy, without sacrificing speed in comparison to existing *de novo *assemblers.

## Background

In contrast with traditional Sanger methods, second-generation sequencing technologies, such as Roche/454 and Illumina/Solexa, produce millions of genome fragments as short DNA sequence reads ( < ~150 bp for Illumina, and < ~500 bp in length for 454, currently). Entire genomes are reconstructed from such fragmented data through a computational process called genome assembly [1]. The most common approaches for solving this problem (Overlap-Layout-Consensus, and the *de Bruijn *graph) first construct a graph encoding the relationships between the sequencing reads generated during the shotgun sequencing process. For the Overlap-Layout-Consensus [2-5], and the related string graph approach [6,7], each node of the graph represents a sequencing read in the input and an edge connects two nodes if the corresponding sequences 'overlap' (the prefix of one sequence matches the suffix of the other with sufficient similarity). In the *de Bruijn *graph approach [8-16], the nodes of the graph are sub-strings of length *k *(*k-*mers) and the edges link together *k-*mers that overlap by exactly *k *- 1 bp only if the *k *+ 1 bp sequence obtained by joining the adjacent nodes is present in at least one of the sequences in the input.

As we will describe in more detail below, irrespective of the approach, computational representations of the resulting graphs require large amounts of memory, thereby requiring substantial computational resources (both memory and run time) to assemble large genomes (such as human). Typical memory requirements for modern assemblers range in the hundreds of giga-bytes (GB) for human genome assembly. Recently, several methods were aimed at reducing the memory requirement of *de novo *genome assembly. In [17], the authors proposed a highly-compressed bitmap representation of a *de Bruijn *graph that can be queried for the existence of individual edges. With this succinct data structure, they were able to reduce the memory consumption by a factor of ~10 compared to common *de Bruijn *graph structure. In [7] the authors relied on read compressed text data structures (the FM index) to construct, on the fly, an assembly string graph. In this paper, we propose an alternative approach to reduce memory usage which exploits the idea of sparseness in genome assembly. Specifically, instead of storing every single *k-*mer (in a *de Bruijn *graph) or read (in an overlap graph) as nodes, we store a sparse subset of these nodes while still ensuring the assembly can be performed. Here, we demonstrate that this approach greatly reduces computational memory demands without sacrificing the accuracy of assembly.

### Memory usage of graph-based assembly paradigms

To introduce concepts central to the approach implemented in *SparseAssembler*, we will briefly discuss the main assembly paradigms and their corresponding memory usage.

### Overlap-Layout-Consensus and string graphs

As briefly outlined above, in the OLC paradigm, the graph contains the reads as nodes, and the edges indicate that the reads overlap. For a given genome coverage *c *(the average number of reads covering a particular base in the genome) for every read, this approach, thus, requires storing approximately *c *overlaps, each of which requires storing a 4-8 byte pointer, as well as at least another 2 bytes of additional information about the overlap (coordinates within the reads, level of similarity, etc.) If we take into account that each read must also record its sequence and possible quality value, we also require an additional 2-8 bits of information per base-pair per read. Storing this graph for a typical human genome sequenced with reads of length 100 at a coverage of 50, requires between ~300-900 GB of memory. Note that in this analysis we omit repeats and errors, both of which further increase the memory requirement.

**Figure 1:**
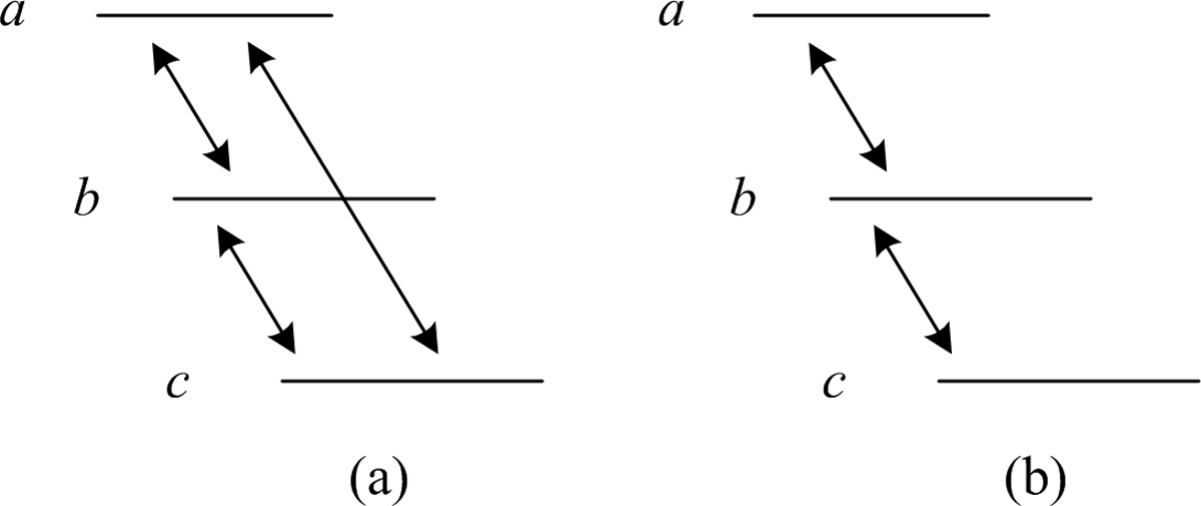
**From overlap graph to a string graph**. (a) an overlap graph, in which all the overlaps are recorded. (b) the string graph, transitive overlap (*a, c*) is removed.

The string graph approach dramatically reduces the memory requirement by a factor roughly proportional to the depth of coverage. A string graph is an overlap graph where transitive edges have been removed, specifically if read *A *overlaps reads *B *and *C*, and *B *also overlaps *C*, (Figure 1a) the overlap (*A, C*) is removed from the graph as it can be inferred from the overlaps between (*A, B*), and (*B, C*) (Figure 1b). As a result, each read only needs to store roughly one overlap (multiple overlaps may need to be recorded due to sequencing errors and repeats), reducing the theoretical memory requirement to roughly 6-18 GB of memory. On real data, a recent assembler relying on the string graph approach was reported to use 54 GB memory for human genome assembly [7].

### *de Bruijn *graph based assembly

**Figure 2:**
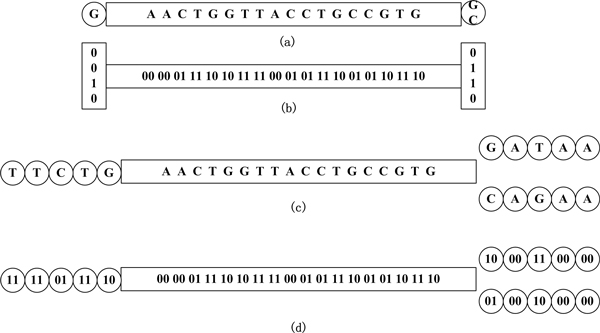
**A node with branches in the *de Bruijn *graph and the sparse *k-*mer graph**. (a) A node with branches in a *de Bruijn *graph. (b) The binary implementation of (a). (c) A node with branches in a sparse *k-*mer graph. (d) The binary implementation of (c). The *k-*mers which are nodes in the graph are squared in the blocks. Neighbouring nucleotides indicating the edges of the graph are circled.

In the *de Bruijn *graph, edges can be implicitly represented by saving only the presence of the neighbouring nucleotides (at most 4 for each *k-*mer). A common first stage of *de Bruijn *graph-based *de novo *assemblers is to build the graph by storing all the *k-*mers and their neighbouring nucleotide(s). A *k-*mer is considered being different only in orientation with its reverse complement, and only one of the two (chosen by lexical-order) is saved. Let all *k-*mers be encoded in bits: 00, 01, 10, 11, respectively, for A, C, G, T, and let 4 bits be used to indicate the presence/absence of the 4 possible edges/nucleotides on every side (Figure 2a, b). Thus, each *k-*mer uses 2 × *k *+ 4 × 2 bits of memory, and the minimum space requirement S_1 _for a genome of size *g *is approximately S_1 _= G × (2 × *k *+ 4 × 2), assuming no additional information needs to be saved. Note that this number does not, in theory, depend on depth of coverage (a *k-*mer is only stored once irrespective of how many reads contain it). However sequencing errors add a huge number of false *k-*mers, thus extra space has to be used to reach successful assembly.

Typically, *k-*mer sizes of 21~51 bp are used because shorter *k-*mers result in branching, and therefore, in ambiguity in the assembly. As a consequence, the memory space required for saving all *k-*mers can be huge. Using traditionally techniques, over 300 GB memory can be used even with a small *k-*mer size of 20 [9], and it is common to use over 100 GB memory even with error-corrected reads with few false *k-*mers [13]. Recent advances in *k-*mer counting (e.g., *Jellyfish *[18] and *BFcounter *[19]) can help improve the memory requirements of *de Bruijn *graph construction. The approach we describe below targets the actual information stored in the graph, thus allowing further memory reductions beyond those achieved by the aforementioned tools.

### Sparse assembly graph

The approach we propose here involves skipping some fraction of the *k-*mers or reads, thus reducing the size of the overall assembly graph necessary to capture the information. Using the example above from the OLC graph, instead of storing overlaps *(A, B*) and (*B, C*), we could simply store overlap (*A, C*) and eliminate read *B *from the graph. In the *de Bruijn *graph, we simply store only one out of every *g *(*g *<*k*) *k-*mers, attempting to subsample as evenly across the original graph as possible. As a result, the size of the *de Bruijn *graph is reduced by a factor of approximately *g *(see full details in materials and methods).

We would like to note that our approach is similar in spirit to the *minimizer *idea introduced by Roberts *et al. *[20]. Their approach is targeted at the task of detecting *k-*mers shared between different reads. The 'traditional' indexing approach requires storing all *k-*mers within a read. Roberts *et al. *[20] propose only storing the lexicographically smallest *k-*mer (*minimizer*) in a size *w *detection window. They noted that the number of *minimizers *is generally smaller than the number of all *k-*mers (consecutive *k-*mers often share a same *minimizer*), and the memory requirement is further reduced by storing the smaller-sized *minimizers *and by using a large detection window. Our approach for simplifying the *de Bruijn *graph is similar in spirit with the *minimizer *approach, as we only store a sparse sub-sample of the *k-*mers found in the reads. Our choice of the *k-*mers stored in the graph attempts to approximate a uniform *k-*mer sampling of the genome, rather than based on lexicographic ordering. In future research we plan to explore the relative benefits of the two approaches, by including lexicographic information within the sparse *k-*mer selection process.

## Methods

### Moving to the sparse *k-*mer graph

In the sparse *k-*mer graph structure, the nodes in the graph represent a 1/*g *subsample of the *k-*mer diversity in the entire genome. The resulting graph differs from the typical *de Bruijn *graph by having longer links, i.e. more nucleotides per branch (see Figure 2c). With the sparsely spaced nodes, the memory requirement for constructing the sparse *k-*mer graph can be considerably less than that for building *de Bruijn *graphs.

Our subsampling procedures proceeds as follows: let *g *equal the number of base pairs skipped between *k-*mers that are stored from each sequencing read. In the ideal case, with no branches and assuming that the *k-*mers are staggered by *g *= 5 bases, we can store ≤ 5 neighbouring bases on each side of the *k-*mer. Although we store more information for each *k-*mer by also extending its links, we store many fewer *k-*mers than currently implemented approaches. More precisely, the total memory space requirement can be calculated as, $S_{2}\approx \frac{N}{g}\times \left(2\times k+2\times 2\times g+\text{ptr\_sz}\right)=N\times \left(\frac{2\times k}{g}+2\times 2+\frac{\text{ptr\_sz}}{g}\right)$ where *ptr_sz *is the extra space required by the pointer structures for the edge links. Compared with the *de Bruijn *graph approach, we reduce *k-*mer storage to 1/*g*, and the portion for storing edges to a half, but add a new space requirement for storing edge links, which requires 2 × g bits for each side of the *k-*mer. In our experiments, we found that using *g *= 10 - 25 was effective.

Interestingly, the lower bound of memory space usage for the sparse *k-*mer graph will decrease as sequencing read length increases and more information is stored in the links. Let reads be of length *r*; the sparse *k-*mers scheme becomes more efficient when *r-k *is large. In this case we can use large *g*'s and still retain sufficient information, which will become more common with the future improvements in sequencing technology. Following this trend of technology advances, we can also increase *k *to larger values than those used currently while still keeping memory usage low.

As a detailed example, we explain how a sparse *k-*mer graph can be constructed. We divide the process into two rounds. In the first round, we select the *k-*mers that will be used as nodes. For each sequencing read, we first query whether any of the subsequent *g k-*mers has already been used as a node. If so, we begin our sub-sample of the read from that node. Otherwise, we select the first *k-*mer as a new node. After the first scan, the nodes are selected, and they are expected to be nearly *g-*gapped if there are no sequencing errors. In real data, we filter out the low-coverage nodes before we move to the second round. Low-coverage nodes are regarded as nodes in spurious branches such as tips or bubbles or real *k-*mer nodes connected to a spurious branch. In the second round, the links between the remaining nodes are built. The accurate coverage of the *k-*mer nodes is recalculated in this round. After two rounds of processing, the *k-*mers picked as nodes are approximately, though not strictly *g-*gapped, which results in some redundancy in space.

To show the effect of *g *on memory storage during assembly, we conducted two simple comparisons using simulated 30× error free 100 bp reads from the fifth chromosome of the *Saccharomyces cervisiae *genome (NC_001137.2), which is around 600 kbp long. First, without any sequencing error, the number of selected nodes, with *k *= 31, *g *= 16, is 36,932. If we set *g *= 1, meaning no skipping, we observe 566,045 nodes in the graph, indicating a 15.3 fold reduction in the size of the graph, which does approximate 1/*g*. When we introduce a uniform 1% error rate into our sequence data, we now obtain 830,309 nodes, with *k *= 31 and *g *= 16, but the full graph also gets larger with the error rate, now containing 12,494,172 nodes, with an effective reduction by 15.0 for *g = 16*. Assembly time and results are also comparable to existing *de novo *assemblers, but a sparse approach greatly reduces the memory requirements and *g *can be adjusted according to the local computing environment. Like in the *de Bruijn *graph the complexity of the graph depends on the value chosen for *k*. The larger *k *is, the less complex the graph. Since the sparse graph encodes the same branches as the original *de Bruijn *graph, the conversion to a sparse graph reduces the memory requirement but does not increase the overall complexity of the resulting structure.

In our experiments, we found that different values for *g *lead to only a slight difference in assembly results, thus, setting *g *to 10~15 provides substantial memory saving without sacrifice in quality. Although increasing *g *should cause us to miss some of the true links due to sequencing error, bias, and low-coverage, we found the assembly quality, measured by corrected NG50 and contig mean size, is usually slightly improved with increasing *g*. Also usually the assembly is better with a larger *g *because many of the short repeating *k-*mers are not saved as nodes, and are only implied by the edge links. The larger the *g*, the fewer repeats are saved.

Last, if two reads overlap by *k + g *bases, an overlap between these 2 reads is also found with the sparse *k-*mer graph and there will be at least one *k-*mer selected in each read. And if two reads share one specific *k-*mer that has been saved in round 1, then the overlap can also be found. Thus, the ability of encoding overlaps between reads within the sparse *k-*mer graph is between using that of *de Bruijn *graphs constructed with *k-*mer sizes between *k *and (*k+g)*. Practically we found using *g = *10~15 provides a good balance point on real datasets.

Note that the graph construction procedure outlined above is order-dependent, i.e., different read orderings will result in a (slightly) different choice of the sparse *k-*mers selected within the graph. In our tests, this non-deterministic behaviour does not seem to affect the performance of the algorithm (either in terms of memory requirement or accuracy), however we plan to explore in the future the use of the *minimizer *concept (described in the Background) to ensure determinism in graph construction.

### Circumventing sequencing errors and graph simplification

**Figure 3:**
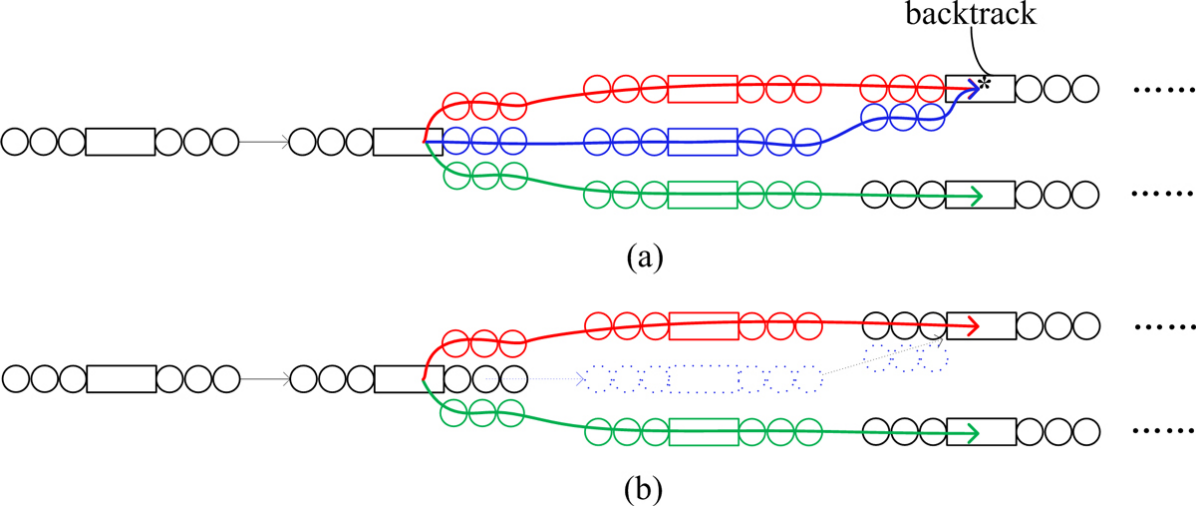
**Breadth-first search bubble removal in the sparse *k-*mer graph**. Removing unwanted structures in the sparse *de Bruijn *graph. (a) Before removal. (b) After removal.

Sequencing errors and polymorphisms can result in tips or bubbles [8] in the assembly graphs, irrespective of the underlying paradigm (*de Bruijn*, sparse *k-*mer, or overlap). To remove these unwanted structures, we first remove the low-coverage nodes and edges. After that, like in *Velvet *[8], we developed a Dijkstra-like breadth-first search algorithm to detect bubbles and tips, using the distance in bp to traverse the branches from near to far. The search backtracks to the last branching node upon reaching a visited node or a tip end. Upon a bubble we choose the higher-coverage branch and remove the weaker branch. Tips are directly removed. After this step, spurious paths and redundant structures like tiny loops and bubbles are removed (Figure 3).

### Genome assembly

The full assembly process consists of (*i*) building the sparse assembly graph as described above and (*ii*) graph simplification and traversal. The procedure for reconstructing the genome is similar to that used in the *de Bruijn *and string graph algorithms. A new traversal begins at a node not visited in previous traversals, and breaks when branches are detected; the separate traversals form the individual contigs reported by the assembler.

## Results

Recent comparisons of assembly software [21-23], including *SSAKE, VCAKE, Euler-sr, Edena, Velvet, ABySS, SOAPdenovo*, and *ALLPATHS *failed to discover significant differences in the magnitude of memory usage, which were all large: they all require > 100 GB memory on human genome assembly even with error free data. We therefore compare our results with only three major state-of-the-art and purely *de Bruijn *graph based assemblers: *ABySS, Velvet*, and *SOAPdenovo*.

To test the sparse assembly idea we implemented a single threaded program *SparseAssembler*, based on the sparse *k-*mer graph and assembly process described above. In all tests, we set assemblers to single end single threaded mode.

In simulated comparisons (Tables 1, 2), we uniformly sampled 30× 100 bp reads from the fruit fly (X, NC_004354.3; IIL, NT_033779.4; IIR, NT_033778.3; IIIL, NT_037436.3; IIIR, NT_033777.2; IV, NC_004353.3) and rice genomes http://rgp.dna.affrc.go.jp/J/IRGSP/Build3/build3.html and introduced uniformly distributed errors at 0.5% error rate and assembled using *k *= 31 for all assemblers and used *g *= 15 for *SparseAssembler*. We chose a somewhat lower error rate than commonly encountered in practice (although after quality trimming and error correction real datasets can achieve such low error rates) in order to allow us to execute all the assemblers being compared. High levels of error lead to increased memory requirements for the majority of existing genome assemblers. We also simulated 50× 200 bp reads (error free as well as 1% error rate) to test the performance using various *k-*mer sizes on a human genome (NCBI build 39, Table 3). We used *k *= 31, 63, 127, and fixed *g *= 25, corresponding to the skipped intermediate *k-*mers. Our simulations on the human genome with varying *k *highlights several interesting phenomena. Limited by memory and read length, current assemblers usually use small *k-*mer sizes (21~64) to assemble human genomes, our simulations suggest longer read lengths could lead to drastic improvements in human genome assembly (Table 3). Longer reads can be obtained with current technology, e.g., through the use of overlapping paired-end reads, currently available [11,24].

**Table 1:** Assembly performance comparison on the fruit fly genome

( *k *= 31 )	*ABySS*	*Velvet*	*SOAPdenovo*	*SparseAssembler*
Time (hr)	5.5	3	3	1
Memory peak (GB)	46	31	14	2
> 100 bp (# contigs)	23,992	23,104	20,580	20,429
Sum (kbp)	113,580	113,574	112,395	113,650
Mean size (bp)	4,734	4,916	5,461	5,563
N50 (bp)	18,317	19,576	25,461	28,355
N95 (bp)	66	61	67	74
Corr NG50 (bp)	18,317	19,576	25,461	28,355
Corr NG95 (bp)	0	0	0	0
Longest contig (bp)	162,263	190,104	195,709	273,977
Coverage (%)	96.24	96.82	95.53	97.83
Misjoins	0	6	0	0

The performance on the fruit fly genome dataset, genome size: 120,291 kbp. Programs are run using default settings.

**Table 2:** Assembly performance comparison on the rice genome

( *k *= 31 )	*ABySS*	*Velvet*	*SOAPdenovo*	*SparseAssembler*
Time (hr)	13	7	16	5
Memory peak (GB)	69	51	29	4
> 100 bp (# contigs)	458,456	397,252	444,545	386,604
Sum (kbp)	253,708	225,618	258,106	262,988
Mean size (bp)	553	568	581	680
N50 (bp)	538	310	655	734
N95 (bp)	38	0	40	31
Corr NG50 (bp)	538	310	655	733
Corr NG95 (bp)	38	0	0	0
Longest contig (bp)	23,220	23,939	26,869	26,890
Coverage (%)	69.2	62.3	71.3	71.5
Misjoins	1	34	10	9

The performance on the rice genome dataset, genome size: 370,733 kbp. Programs are run using default settings.

**Table 3:** Assembly performance on the human genome

	*k *= 31	*k *= 63	*k *= 127	*k *= 31	*k *= 63	*k *= 127
	**Error free data**			**1% error rate**		

Memory peak (GB)	14	16	19	30	49	51
> 100 bp (# *k *contigs )	3,195	1,984	714	2,727	1,554	1,359
Sum (G bp)	2.37	2.79	2.83	2.29	2.72	2.88
Mean size (bp)	743	1,406	3,961	839	1,751	2,121
N50 (bp)	2,130	6,479	79,906	2,121	6,319	49,572
N90 (bp)	244	631	10,441	304	872	1,021
Longest contig (bp)	50,800	124,293	801,692	47164	124,292	537,017

To test performance on real data, we compared our approach on 100-bp-read whole-genome shotgun sequence data generated on the Illumina platform for *Escherichia coli K-*12 MG1655 (NCBI SRA accession ERR022075), human chromosome 14, and a whole human genome (NA12878). The performance on some other real datasets (including single cell reads and *Ion Torrent PGM *reads) can be found on our website. For the *E. coli *dataset, *k *= 51 was used for all assemblers (*SOAPdenovo *did not output reasonable results on this dataset, so we did not include the result, with N50 ~ 200), and we set *g *= 15 for *SparseAssembler *(Table 4), for the human chromosome 14, *k *= 53 was used for all assemblers, and we set *g *= 15 for *SparseAssembler *(Table 5), and *k *= 31-51, *g *= 25 for the whole human genome (Table 6). On these real datasets from the Illumina platform, our approach used around 1/10 memory compared with other assemblers and produced comparable results (Tables 4, 5). Because the bubble merging strategy in *SparseAssembler *is simple, the results on real data can include more misjoins than other assemblers but these misjoins appear to occur within the shorter contigs, thus achieving a corrected NG50 not much different from the original NG50 size (Tables 4, 5). The corrected assembly statistics are obtained by fragmenting the assembly wherever errors are encountered in the data. The runtime of *SparseAssember *was smaller than that for other assemblers. Raw Illumina reads (80X, length 100) for a member of CEU HapMap population (identifier NA12878) sequenced by the Broad Institute were downloaded from ftp://ftp.1000genomes.ebi.ac.uk/vol1/ftp/technical/working/20101201_cg_NA12878/NA12878.hiseq.wgs.bwa.raw.bam in the last test. This dataset was also used in [7] and 54 GB memory was consumed to first clean the reads before assembly, using just half of the reads in the dataset. For testing purpose, we also used 40× reads (the first end of the paired library). The most expensive assembly with uncleaned reads took 29 GB memory and roughly 1 day resulting in an N50 size of 2,915 and assembled length of 2.70 G. All runs were single-threaded. Though our quality is lower than some assemblers, using corrected reads and mate-pair information in the future is expected to further improve the assembly result. Most other assemblers take hundreds of GBs of memory which is beyond our computer's reach, but the detailed consumptions on similar datasets can be found in related references.

**Table 4:** Assembly performance on the *E.coli *genome (ERR022075)

(*k *= 51)	*ABySS*	*Velvet*	*SparseAssembler*
Time (hr)	2	1	0.7
Memory peak (GB)	3.5	9.1	0.7
> 100 bp (# contigs)	430	632	485
Sum (bp)	4,556,772	4,413,080	4,577,604
Mean size (bp)	10,597	6,983	9,438
N50 (bp)	57,655	19,067	57,830
N95 (bp)	5,629	128	5,906
Corr NG50 (bp)	57,655	19,067	57,828
Corr NG95 (bp)	5,629	125	5,676
Longest contig (bp)	166,107	120,922	173,976
Coverage (%)	99.90	96.53	99.94
Misjoins	1	1	2

**Table 5:** Assembly performance on the human chromosome 14

(*k *= 53)	*ABySS*	*Velvet*	*SOAPdenovo*	*SparseAssembler*
Time (hr)	6	2.5	6.	1.9
Memory peak (GB)	49	37	30	3
> 100 bp (# contigs)	85,181	129,046	84,719	55,024
Sum (kbp)	88,663	89,854	87,908	86,296
Mean size (bp)	1,041	696	1,038	1,568
N50 (bp)	3,568	1,499	3,117	3,890
N95 (bp)	179	184	197	202
Corr NG50 (bp)	3,475	1,487	3,065	3,760
Corr NG95 (bp)	175	178	192	198
Coverage (%)	98.54	98.86	98.42	97.56
Longest contig (bp)	61,018	16,043	49,584	60,797
Misjoins	24	62	47	61

This dataset was downloaded from http://gage.cbcb.umd.edu/data/index.html, genome size: 88,289,540.

**Table 6:** Assembly performance on the NA12878 human genome

	*k *= 31	*k *= 41	*k *= 51
Memory peak (GB)	26	29	29
> 100 bp (# *k *contigs )	2,740	2,800	2,744
Sum (G bp)	2.33	2.57	2.70
Mean size (bp)	743	919	3,961
N50 (bp)	2,054	2,647	2,915
N95 (bp)	318	335	380
Longest contig (bp)	36,460	38,864	50,441
Corr NG50 (bp)*	1,502	2,213	2,610
Corr NG95 (bp)*	0	0	114
Misjoins*	21	19	17

* The corrected statistics are calculated by mapping back to human chromosome 14.

In all comparisons, our sparse *k-*mers based *SparseAssembler *uses substantially less computational memory and completed the assembly in a comparable period of time and with comparable quality with several state-of-the art assemblers. In tests with known reference genomes (Tables 1, 2, 4, 5), the assembled results were mapped back to the known reference genome using *MUMmer3 *[22,25,26] to count the number of misjoins. Contigs that contain false joins were broken into smaller but accurate contigs. The corrected NG50s were calculated based on the size of these smaller contigs. This approach is similar to that used in the GAGE assembly evaluation [22]. We did not map back the assembled whole human genomes because of hardware limitations, instead in Table 6, we map the contigs back to a smaller region, the chromosome 14.

## Discussion and Conclusions

This new sparse graph approach to *de novo *genome assembly, as implemented here in *SparseAssembler*, consistently produces comparable results to the current state-of-the-art *de Bruijn *graph-based assemblers, demands considerably smaller amounts of computer memory, using both simulated and real data. This approach can be extended for a sparse string graph as well, by selecting a sparse subset of the reads when constructing the overlap graph. Future improvements, such as incorporating more efficient data structures, promise to reduce memory demand further. Also the assembly approach used in our paper is a simple implementation resembling the *de Bruijn *graph approach, meant to illustrate the power if this approach, and we expect much better assembly results can be obtained by incorporating our ideas within existing genome assemblers.

In the *k-*mer graph framework, the memory savings achieved by *SparseAssembler *is similar to that achieved with Conway & Bromage's succinct data structure but is simpler in idea and implementation. Moreover, the savings of our assemblers are scalable with the length of *g*. Thus, as read lengths improve, the links between *k-*mers can be extended and the graph can become even sparser, reducing memory demands as sequencing technology develops. Finally, the sparse *k-*mer graph shares all advantages of the *de Bruijn *graph model.

Therefore, the results reported here strongly support our idea that a sparse assembly graph retains sufficient information for accurate and fast *de novo *genome assembly of moderate-size genomes in a cheap, desktop PC computing environment, which is usually only equipped with several gigabytes memory. Future improvements to *SparseAssembler *will focus on extending this approach to a sparse string graph and the exploitation of paired-end reads.

## Availability

Related programs and code are available at:

http://sites.google.com/site/sparseassembler/.

## Competing interests

We declare that we have no significant competing financial, professional or personal interests that might have influenced the performance or presentation of the work described in this manuscript.

## Authors' contributions

CY developed the algorithms, collected results, and wrote the software. ZM, CC, MP, DY contributed discussions on algorithms. CY, ZM, CC, MP, and DY wrote the manuscript. All authors read and approved the final manuscript.
